# Review no. 1: designing clinical kidney research using real-world data: research questions, data sources, and analytical skills

**DOI:** 10.1007/s10157-025-02789-3

**Published:** 2025-12-09

**Authors:** Yuka Sugawara, Masao Iwagami, Hajime Nagasu, Yoshihisa Miyamoto, Megumi Oshima, Takashige Kuwabara, Tadashi Sofue, Naoki Nakagawa

**Affiliations:** 1 Division of Nephrology and Endocrinology, The University of Tokyo, Tokyo, Japan; 2 Subcommittee for the Promotion of Clinical Research, The Japanese Society of Nephrology, Tokyo, Japan; 3 Department of Digital Health, Institute of Medicine, University of Tsukuba, Tsukuba, Japan; 4 Department of Nephrology and Hypertension, Kawasaki Medical School, Kurashiki, Japan; 5 Department of Real-World Evidence, The University of Tokyo, Tokyo, Japan; 6 Department of Nephrology and Rheumatology, Kanazawa University, Kanazawa, Japan; 7 Department of Nephrology, Kumamoto University Graduate School of Medical Sciences, Kumamoto, Japan; 8 Department of Cardiorenal and Cerebrovascular Medicine, Faculty of Medicine, Kagawa University, Takamatsu, Japan; 9 Division of Cardiology and Nephrology, Department of Internal Medicine, Asahikawa Medical University, Midorigaoka-Higashi 2-1-1-1, Asahikawa, Japan

**Keywords:** Real-world data, Electronic health records, Chronic kidney disease, Acute kidney injury, End-stage renal disease

## Abstract

This review series provided methodological guidance for clinical kidney research using real-world data, building on the “Hands-on R Seminar for Clinical Research: acute kidney injury (AKI) Detection and estimated glomerular filtration rate (eGFR) Slope Estimation from Creatinine Data,” held at the 68th Annual Meeting of the Japanese Society of Nephrology in 2025. The seminar offered participants mock datasets, R scripts, and practical exercises to set up analysis environments and conduct data analyses, alongside brief lectures on conducting clinical research on AKI and eGFR decline. This series expands and complements the seminars. In Part 1, we provide an overview of the key components essential for successful clinical kidney research. First, formulating a robust research question is crucial, grounded in clinical experience and informed by up-to-date evidence. Common outcomes or exposures in clinical kidney studies include eGFR slope (as a marker of chronic kidney disease progression), AKI incidence, and initiation of kidney replacement therapy. Second, identifying appropriate data sources is necessary. In addition to primary data collection, routinely collected electronic health records and real-world databases (including disease registries) have become more accessible. Here, we summarize real-world databases in Japan, particularly those that include serum creatinine and urine test results. Finally, researchers require proper data handling and analytical skills. We highlight kidney research-specific techniques, such as AKI detection and eGFR slope calculation from longitudinal creatinine data. Subsequent articles in this series (Part 2 and beyond) will detail each specific method and include practical R commands.

## Introduction

In nephrology practice, patients routinely undergo blood and urine testing, particularly serum creatinine and urinary albumin, for the diagnosis and/or follow-up of kidney diseases. These data are continuously generated in hospitals and other healthcare facilities as part of daily patient care and may also be collected during community health checkups. Although primarily intended to guide clinical decision-making for individual patients, such data can also serve as valuable resources for research.

We are currently in an era of abundant data, where clinical research is no longer limited to academic institutions. Clinicians in general practice can also actively engage in clinical research activities. In nephrology, the number of clinical trials remains relatively low compared with other medical specialties, and treatment options for kidney diseases remain limited [1–3]. However, as more clinicians become involved in research and the base of clinical research expands, new insights are likely to emerge, ultimately contributing to improved care and outcomes for patients with or at risk of chronic kidney disease (CKD) and acute kidney injury (AKI).

We have proposed three essential elements to summarize the key components of well-conducted clinical studies: (1) a well-defined research question, (2) an appropriate dataset to address the question, and (3) analytical skills to handle and interpret the data. Together, these three elements form the foundation of meaningful clinical research. Early-career researchers may begin with small datasets available at their institutions. However, such initial experiences often lead to new opportunities and the development of further research ideas.

To support clinicians and researchers—particularly those with limited experience—in conducting clinical kidney research, the Japanese Society of Nephrology’s subcommittee for the promotion of clinical research recently organized a hands-on seminar, titled “Hands-on R Seminar for Clinical Research: AKI Detection and eGFR Slope Estimation from Creatinine Data,” at the 68th Annual Meeting of the Japanese Society of Nephrology in 2025, providing practical tips, R scripts, and mock datasets to enable participant perform data analysis on their computers. This review series builds on that seminar, expanding its explanations to complement the training materials.

This article, the first of the review series, aimed to provide an overview of all three components, including how to formulate a meaningful research question, a list of real-world data (RWD) sources available in Japan, and the analytical skills required for clinical kidney research. Among these skills, several warrant more detailed discussion, such as AKI identification and staging, calculation of the estimated glomerular filtration rate (eGFR) slope from longitudinal creatinine data, and the interpretation of urine test results, such as the urinary albumin-to-creatinine ratio (UACR). These topics will be explored in greater depth in subsequent articles in this series.

## Patterns of research questions in kidney research

We begin with the first key component of clinical research: formulating a clinical research question. Developing a meaningful question required reflecting on the uncertainties and observations that arise in daily clinical practice, ensuring the work addresses real-world clinical needs rather than being research for its own sake. A thorough review of existing literature is also essential to clarify what is already known, avoid redundancy by preventing the investigation of questions that have already been answered, and deepen the understanding of the context surrounding the research topic. In addition, it provides valuable insights into appropriate study populations and methodological approaches.

Furthermore, understanding common patterns of research questions may be helpful. As shown in Table 1, outcomes such as the incidence of AKI or eGFR decline may serve as endpoints in descriptive or analytical studies, exposures in analytical studies, or effect modifiers. In descriptive studies, outcomes such as AKI frequency or the average rate of kidney function decline within a particular population (such as patients with diabetes) are of primary interest. Temporal trends or regional variations are also valuable areas of investigation. In analytical studies with AKI or rapid decline in eGFR as outcomes, researchers are often motivated to identify novel risk factors or compare outcomes across groups. Predicting the future incidence of AKI or rapid decline in eGFR from individual risk factors is another common objective.

**Table 1:** Examples of patterning research questions

	Outcome in a descriptive study	Outcome in an analytical study	Exposure in an analytical study	Effect modifier in an analytical study
AKI incidence	How frequently does AKI occur within a specific population? Does the incidence of AKI vary by time and region?	What are risk factors for AKI within a specific population? Does exposure to drug A (vs. drug B) increase the risk of AKI? Can we predict the incidence of AKI from (known) risk factors of individual patients?	Does the occurrence of AKI influence patient prognoses or the future incidence of other diseases within a specific population?	Does the effect of drug A on cardiovascular outcomes differ by the presence/absence of AKI history?
eGFR decline	What is the average speed of kidney function decline within a specific population? What proportion of patients in a given population exhibit rapid progression of CKD?	What are risk factors for a rapid decline in kidney function within a specific population? Is exposure to drug A (vs. drug B) associated with the slower rate of CKD progression of individual patients? Can we predict rapid decliners from (known) risk factors?	Does the rate of kidney function decline or a rapid decline influence patient prognosis or the future incidence of other diseases within a specific population?	Does the effect of drug A on cardiovascular outcomes differ between rapid decliners and other patients?

*AKI* acute kidney injury; *eGFR* estimated glomerular filtration rate

Note: Many other research questions are not included in this table

Further, in analytical studies with AKI or eGFR slope as exposures, researchers are often interested in the prognosis of patients with AKI or rapid eGFR decline compared with those without. Finally, effect modification (also referred to as heterogeneity or interaction) may be a difficult concept for beginners; however, it provides an important perspective when formulating research questions [4, 5]. For example, whether the effect of an emerging drug (such as sodium-glucose cotransporter 2 inhibitors or mineralocorticoid receptor antagonist) differs between patients with and without a history of AKI, or between those with and without rapid eGFR decline, may be clinically relevant. Notably, other types of research questions, not included in Table 1, may also be explored.

## Potential real-world data sources for kidney research in Japan

Next, we introduce potential RWD sources available for kidney research in Japan, while those from other countries have been described elsewhere [6]. RWD was formally defined by the US Food and Drug Administration as “data relating to patient health status and/or the delivery of health care routinely collected from a variety of sources” [7].

Major RWD sources in Japan, similar to those in many countries [6], include electronic health records (EHR), insurance claims and billing data, drug and disease registries, health check-up data, patient-generated data, including those from home-use applications, and information from social media platforms. Although RWD encompasses various sources, it can be conceptually classified in terms of accessibility: (1) EHR and claims/billing data from a researcher’s own institution, (2) EHR and claims/billing data from institutions within the same hospital network, (3) commercially available EHR and claims/billing data, and (4) non-commercial RWD, including disease registries, biobanks, and cohort studies. Recent disease registries and cohort studies often include both automatically collected data (EHR or claims/billing data) and data manually entered by clinical staff or medical information technicians. In addition, data from randomized controlled trials can be used for secondary analyses [8], though regarding this as RWD or not may be controversial.

Table 2 summarizes the key characteristics of the Japanese databases (mainly disease registries) specifically established for kidney diseases, while Table 3 summarizes databases not specifically established for kidney research, but nonetheless applicable to it. Other Japanese databases may exist beyond those included in the tables. The Japan Diabetes Comprehensive database project based on an Advanced Electronic Medical record System (J-DREAMS) [9, 10] is included in Table 2 (rather than Table 3) because its structure is closer to that of J-CKD-DB, and kidney outcomes are among the most important outcomes in patients with diabetes. These databases differ in several important aspects, including data sources (such as EHR vs. claims/billing data), target populations and coverage (such as disease-specific cohorts vs. the general population), and accessibility and usability.

**Table 2:** Example of Japanese databases specifically established for kidney diseases

	J-CKD-DB/ J-CKD-DB-Ex	J-DREAMS	J-KDR/J-RBR	CKD-JAC	JRDR	J-DOPPS
Brief summary	EHR-based database of non-dialysis CKD patients from university hospitals, rich in laboratory data	EHR-based database of patients with diabetes in Japan	Nationwide non-dialysis CKD registry collecting clinical and biopsy data	Non-dialysis CKD registry, rich clinical data with long-term follow-up	The nationwide registry of dialysis patients in Japan covers nearly all maintenance dialysis patients with annual updates	International prospective registry of patients undergoing dialysis, including detailed clinical data and facility practices
Collected data						
Laboratory data at medical institutions	Yes	Yes	Yes	Yes	Yes	Yes
Prescription	Yes	Yes	No	No	Yes	Yes
Administrative claims data	Disease names only	Disease names only	No	No	No	No
Others	–	BH, BW, BP, time of diabetes diagnosis, and diagnosis of complications	Clinical and pathological diagnoses, biopsy information	BH, BW, BP, end-stage kidney disease, CVD events, deaths	Dialysis-related information (schedule, Kt/V, DW), information on facilities, BP, CVD events, and deaths	Dialysis-related information (schedule, Kt/V, DW), information on facilities, BP, CVD events, and deaths
Sample size (present)	approx. 520,000	approx. 100,000	approx. 45,000	approx. 3000	approx. 350,000	> 10,000 in total
Data source paper	Nakagawa et al. [11]	Sugiyama et al. [9]	Ozeki et al. [15]	Imai et al. [16], Hamano et al. [17]	Hanafusa et al. [18]	Young et al. [20]
Website URL	https://j-ckd-db.jp/	https://www.j-dreams.jihs.go.jp/	https://jsn.or.jp/member/registry/	https://center6.umin.ac.jp/cgi-open-bin/ctr/ctr_view.cgi?recptno=R000023138	https://www.jsdt.or.jp/dialysis/2227.html	http://www.jinzouzaidan.or.jp/j-dopps/index.html
Examples of research papers	Sofue et al. [54], Nagasu et al. [55]	Sugawara et al. [56], Yamada et al. [57]	Nakagawa et al. [58], Oda et al. [59]	Inaguma et al. [60], Imaizumi et al. [61]	Sugawara et al. [62], Kikuchi et al. [63]	Okada et al. [64], Kato et al. [65]

*BP* blood pressure; *CKD* chronic kidney disease; *CKD-JAC* Chronic Kidney Disease Japan Cohort, *CVD* cardiovascular disease; *DW* dry weight; *EHR* electronic health record; *J-DOPPS* Japan Dialysis Outcomes and Practice Patterns Study; *J-DREAMS* Japan Diabetes Comprehensive Database Project based on an Advanced Electronic Medical Record System; *J-KDR* Japan Kidney Disease Registry; *J-RBR* Japan Renal Biopsy Registry; *J-RDR* Japanese Society for Dialysis Therapy Renal Data Registry

Note: J-DREAMS is included in Table 2 (instead of Table 3) because the structure of J-DREAMS is closer to that of J-CKD, and kidney outcomes are one of the most important outcomes in patients with diabetes

**Table 3:** Example of Japanese databases not specifically established for kidney diseases, but potentially useful for kidney research

	NDB	DPC	MID-NET	MDV	JMDC (Hospital-based)	JMDC (Payer/claims-based)	DeSC Healthcare
Brief summary	Claims database covering the entire insured population of Japan; however, it lacks laboratory test results except for checkups	Nationwide inpatient claims database developed for acute care hospitals participating in the DPC/per-diem payment system	EHR-derived database integrating clinical data, including laboratory values and prescriptions from multiple hospitals	Commercially available hospital-based database combining claims and partial laboratory data from DPC hospitals	Commercially available hospital-based EMR-linked database with detailed clinical information from participating institutions	Commercially available claims database with annual health checkup data for working-age individuals and their families	Commercially available claims database with health checkup data for working-age individuals and their families, and older individuals aged ≥ 75 years from various insurers
Collected data							
Laboratory data in clinical practice	No (except for checkups)	No	Yes	Yes (from DPC hospitals)	Yes	No (except for checkups)	No (except for checkups)
Prescription	Yes	In-hospital only	Yes	In-hospital only	Yes	Yes	Yes
Administrative claims data	Yes	Yes	Yes	Yes	Yes	Yes	Yes
Others	Specific health checkups in the community (including serum creatinine and urine tests)	Discharge summaries, surgical procedures, and diagnosis-related grouping data	Vital signs, disease registry linkage, limited to participating hospitals	DPC-based discharge summaries, test orders, and partial outcome data	Vital signs, EMR-based clinical notes (varies by hospital)	Health checkup data (including BP, BMI, serum creatinine, and urine tests)	Health checkup data (including BP, BMI, serum creatinine, and urine tests)
N	approx. 120 million (the entire Japanese population)	approx. 10 million/year	approx. 8 million	approx. 40 million	Approx. 5 million	approx.14 million	approx. 10 million
Target population	Everyone covered by public insurance (all ages)	Inpatients in acute care hospitals under the DPC system	Hospital in/outpatients at participating centers	Patients in DPC hospitals	Patients in participating hospitals with EMR	Working-age individuals and dependents under corporate insurance	Corporate health insurance enrollees (Kenpo) and enrollees of the National Health Insurance (Kokuho), and the Advanced Elderly Medical Service System
Cross-institutional patient linkage	Yes	No	Limited, hospital-based	No	No	Yes	Yes
Data source paper	Yasunaga et al. [24]	Yasunaga et al. [34]	Yamaguchi et al. [29]	Laurent et al. [25]	Nagai et al. [32]	Laurent et al. [25]	Yasunaga et al. [26]
Website URL	https://www.mhlw.go.jp/ndb/opendatasite/	https://www.mhlw.go.jp/stf/seisakunitsuite/bunya/kenkou_iryou/iryouhoken/dpc/	https://www.pmda.go.jp/safety/mid-net/0001.html	https://en.mdv.co.jp/ebm/about-mdv-database/	https://www.phm-jmdc.com/hospital-database	https://www.jmdc.co.jp/jmdc-claims-database/	https://desc-hc.co.jp/en
Examples of research papers	A. NDB open data Oda et al. [66] Suzuki et al. [67] B. NDB full data Kubo et al. [68] Li et al. [69]	Miyamoto et al. [70], Okada et al. [71], Watanabe et al. [72]	Hasegawa et al. [73], Waki et al. [74]	Sato et al. [75], Kawai et al. [76]	Mitsuboshi et al. [77], Suzuki et al. [78]	Kimura et al. [79], Iwagami et al. [80]	Okada et al. [81], Moriyama et al. [82], Nakayama et al. [83], Kobayashi et al. [84]

*BP* blood pressure; *BMI* body mass index; *DPC* diagnosis procedure combination; *EHR* electronic health record; *JMDC* Japan Medical Data Center; *MHLW* Ministry of Health, Labour and Welfare; *MDV* Medical Data Vision MID-NET;, Medical Information Database Network; *NDB* National Database of Health Insurance Claims and Specific Health Checkups of Japan; *PMDA* Pharmaceuticals and Medical Devices Agency

As shown in Table 2, J-CKD-DB and J-CKD-DB-Ex [11–14] focus on patients with CKD characterized by an eGFR < 60 mL/min/1.73 m^2^ or the presence of proteinuria. These databases include detailed information on kidney function, urinalysis, and parameters related to CKD-associated anemia and mineral and bone disorders. Both were established with the support of the Japanese Society of Nephrology as multicenter CKD clinical databases derived from EHRs of university hospitals across Japan using the SS-MIX2 standardized format. The J-CKD-DB, initiated in late 2014, contains cross-sectional data, including laboratory results from inpatient and outpatient settings, diagnostic codes, prescription records, and hospitalization information. The J-CKD-DB-Ex is an expanded version that incorporates follow-up data, enabling longitudinal analyses of CKD progression and management.

J-DREAMS [9, 10], launched in 2015 through a collaboration between the National Center for Global Health and Medicine (currently the Japan Institute for Health Security) and the Japan Diabetes Society, is a nationwide EHR-linked registry of patients with type 2 diabetes. It serves as a valuable resource for real-world outcome analyses of diabetes treatment and for studying comorbid conditions, such as diabetic kidney disease. The Japan Kidney Disease Registry/the Japan Renal Biopsy Registry (J-KDR/J-RBR) [15] is notable for its inclusion of kidney biopsy-related data, making it a valuable resource for studying histopathological classifications and their association with clinical outcomes in various glomerular diseases. The Chronic Kidney Disease Japan Cohort (CKD-JAC) [16, 17], although smaller in scale than the aforementioned databases, offers detailed clinical information and long-term follow-up data on patients with non-dialysis-dependent CKD, enabling in-depth analyses of disease progression, risk factors, and cardiovascular comorbidities. The Japanese Society for Dialysis Therapy Renal Data Registry (JRDR) [18, 19] covers nearly all dialysis patients in Japan and is a nationally representative dataset that enables research on patient demographics, treatment practices, survival outcomes, and dialysis-related complications. The Japan Dialysis Outcomes and Practice Patterns Study (J-DOPPS) [20–22] contributes internationally harmonized data, facilitating comparisons of dialysis care and outcomes across multiple countries, and has been instrumental in identifying best practices in hemodialysis.

In Table 3, the administrative claims databases, including the National Database of Health Insurance Claims and Specific Health Checkups of Japan (NDB) [23, 24], Japan Medical Data Center (JMDC) Payer database [25], and DeSC Healthcare database [26, 27], are primarily constructed from billing and insurance claims data. These datasets are based on coded diagnoses, procedures, and prescriptions, and are widely used for health services and epidemiological research. In addition, community residents in Japan are encouraged (those aged 40–74 years are mandated) to attend annual health checkups held at clinics affiliated with companies or public health centers. Although serum creatinine and urine measurements are not always included, they are routinely collected in some centers and can thus be leveraged for CKD research.

Hospital-based databases, such as the Medical Information Network (MID-NET) [28, 29], Medical Data Vision (MDV) [25, 30], and JMDC Hospital databases [31, 32], collect claims and EHR data, including laboratory values (such as serum creatinine and urine protein) from both outpatient and inpatient encounters. These databases are available for AKI and CKD research; however, caution is needed, as data are only captured when patients seek care at participating hospitals. For example, AKI status/stage may be misclassified owing to the lack of outpatient serum creatinine measured at clinics or hospitals outside the database [33]. In addition, if patients with a faster or slower decline in eGFR were more likely to seek care outside the database, missing data and/or loss to follow-up may introduce bias.

The Diagnosis Procedure Combination (DPC) database [34] lacks laboratory values, making AKI and creatinine- or proteinuria-based CKD staging impossible; however, research on AKI requiring kidney replacement therapy and end-stage kidney disease is possible. Researchers should recognize that coded diagnoses of AKI and CKD in claims data are generally characterized by high specificity but low sensitivity [35, 36], which may result in misclassification of CKD/AKI status/stage and consequently bias study results if used in isolation.

In addition, the National Clinical Database (NCD) [37], although not listed in Tables 2 and 3, is a large surgical registry that captures detailed information on surgical procedures across Japan. It is particularly valuable for analyzing vascular access surgeries, such as arteriovenous fistula creation, and kidney transplantation practices and outcomes.

The ease of access and usability of databases for researchers vary considerably. At the time of writing, databases such as JMDC (Payer and Hospital), MDV, and DeSC were commercially available and could be accessed by both academic and industry researchers under contractual agreements with data providers. In contrast, access to J-CKD-DB and J-DREAMS is currently limited to participating institutions or members of affiliated academic societies, often requiring internal coordination and approval. Several registry-based databases, including J-KDR/J-RBR, CKD-JAC, JRDR, and J-DOPPS, also have restricted accessibility, requiring formal applications, institutional or academic collaboration, and review by steering committees. These databases typically operate under a collaborative research model, offering rich clinical detail and longitudinal data but limited independent access.

The NDB and DPC databases are publicly managed and require a rigorous application process, strict review, and mandatory disclosure of research results, which may pose practical barriers to entry. The NDB is available in two forms: NDB Open Data and NDB Full Data. NDB Open Data are publicly accessible without application or cost, but provide only aggregated nationwide statistics, limiting individual-level tracking or detailed analyses. These data are useful for monitoring broad trends. For more in-depth longitudinal analyses, researchers must apply to the Ministry of Health, Labour and Welfare for access, which, upon approval and payment of associated fees, allows for detailed patient-level analyses.

Although the number of cases may be limited, data derived from an investigator’s own hospital or affiliated hospital network offer substantial advantages in accessibility and usability. When allowed under ethical review and institutional policies, researchers can select variables of interest and organize them in formats tailored to their study objectives. In this sense, such data may represent the most flexible and user-friendly resources for clinical research.

## Data handling and analysis skills necessary for kidney research

The next key component is the set of skills required to conduct clinical research (Table 4). Although not unique to nephrology, one of the first essential steps in any analysis is to become familiar with the dataset and perform appropriate data cleaning and formatting using relevant software (such as SAS, R, Stata, SQL, and Python), which are crucial for ensuring the accuracy and reliability of subsequent analyses. Notably, for numerical variables, examining their distributions, such as minimum, maximum, mean, and median values, and visualizing them using histograms, is important. For categorical or string variables, researchers should check for unintended or inconsistent entries. This process is particularly important when working with multi-institutional data, where variables recorded in the same column may differ in definition or may be expressed in different units across sites.

**Table 4:** Examples of key analytical skills in clinical kidney research

Key items	Details
Handling of data	Data formatting Checking the distribution of the data Checking (and excluding) unreasonable data Conversion of "long" data into "wide" data and vice versa as needed Calculation of eGFR from serum creatinine Calculation of UACR
Processing of key kidney-related indicators	Flagging AKI status and stage Calculation of eGFR slope (using ordinary least squares regression, or linear mixed-effects model) Calculation of UACR change Flagging other kidney events (e.g., CKD stage progression, 30% or 40% eGFR decline from the baseline, end-stage kidney disease)
Common analytical methods	Descriptive analysis Statistical tests (such as t-test, chi-square test) Regression analysis Survival analysis
Advanced analytical methods	Multiple imputation Propensity score methods G methods to deal with time-dependent covariates Competing risk analysis Discrimination Instrumental variable analysis Machine learning methods etc

*AKI* acute kidney injury; *eGFR* estimated glomerular filtration rate; *ROC* receiver operating characteristic; *UACR* urine albumin-to-creatinine ratio

For kidney research, researchers often need to calculate eGFR from serum creatinine using established equations. Several equations have been developed to estimate the glomerular filtration rate. The most widely recognized are the revised Modification of Diet in Renal Disease (MDRD) [38] and Chronic Kidney Disease Epidemiology Collaboration (CKD-EPI) equations [39], with multiple versions available. The CKD-EPI equation offers greater accuracy and better prognostic ability than the MDRD equation [40–42]. The most recent KDIGO guidelines recommend the use of the 2021 CKD-EPI creatinine equation, which omits the race coefficient for Black individuals [43]. Furthermore, when both serum creatinine and cystatin C levels are available, the combined CKD-EPI equation offers improved precision and risk prediction [43].

However, in Japanese populations, both the MDRD and CKD-EPI equations may overestimate kidney function. To adjust for this, it is recommended to multiply the result by a Japanese coefficient: 0.813 and 0.908 for the 2021 CKD-EPI creatinine-based (eGFRcr) [44] and 2021 CKD-EPI creatinine-based–cystatin C–based (eGFRcr-cys) equations, respectively [45]. These coefficients were originally derived for the 2009 version of the CKD-EPI equation. However, as population-specific coefficients for the 2021 version have not yet been established, previously calculated values are provisionally applied.

The JSN has developed the JSN eGFRcr equation, a modified version of the MDRD equation derived from Japanese patient data, which is now widely used for clinical evaluation [46]. A cystatin C-based equation, JSN eGFRcys, is also available for estimating eGFR in Japan [46]. Current clinical practice guidelines in Japan recommend the JSN eGFRcr equation, which remains the most commonly used formula in routine clinical practice. The Appendix shows the relevant commands for calculating eGFR from serum creatinine using the 2021 CKD-EPI eGFRcr, 2021 CKD-EPI eGFRcr-cys, JSN eGFRcr, and JSN eGFRcys equations in the statistical software R.

Albuminuria and proteinuria assessment is essential in kidney research and clinical practice. Internationally, the UACR is the standard evaluation method; however, in Japan, the urinary protein-to-creatinine ratio (UPCR) remains commonly used owing to reimbursement constraints in the Japanese healthcare system. In clinical practice, initial assessments are often performed using dipstick urinalysis. To harmonize data across different measurement methods, several equations have been proposed to convert qualitative dipstick results or UPCR values into estimated UACR, facilitating the standardization of data in clinical research [47].

**Fig. 1:**
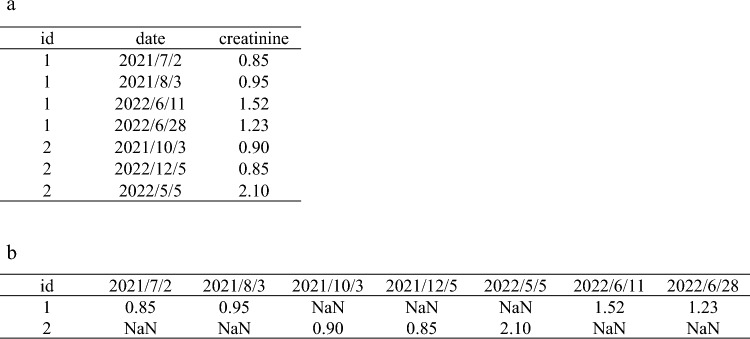
Illustration of transforming data from long to wide format. a) long-format dataset showing longitudinally measured creatinine values for individuals with IDs 1 and 2. b) the same data shown in Fig. 1a, transformed into a wide-format structure

For data analysis, a cleaned dataset can be structured in either a “wide” format, where each row represents one individual, or a “long” format, where each individual may appear in multiple rows to reflect repeated measurements or clinical events (Fig. 1). Although wide-format data are generally easier for beginners to interpret and analyze, longitudinal clinical data, such as those used in nephrology research, are often recorded in long format. Therefore, kidney researchers should be familiar with long-format structures and be able to convert datasets between “long” and “wide” formats as needed. These techniques will be discussed in detail in the next article of this series.

Longitudinal analysis of serum creatinine data enables AKI detection and eGFR slope estimation, a widely accepted surrogate endpoint in CKD research [48–50]. In addition, with the “wide” format data, researchers could flag the timing of CKD stage progression (for example, first observation of CKD stage G3a in a patient previously at stage G2) or the timing of 30% or 40% eGFR decline from the baseline. Furthermore, changes in albuminuria have recently been recognized as a validated surrogate outcome [48, 51, 52].

Regarding subsequent analytical techniques, common approaches in kidney research include multivariate logistic regression, multivariate linear regression, and survival analysis (such as Cox proportional hazards models) (Table 4). Advanced knowledge and techniques, such as competing risk analysis, may be needed; for example, when studying the longevity of arteriovenous fistulas in patients undergoing dialysis with high mortality rates [53]. Researchers interested in advanced methods are encouraged to consult relevant textbooks and publications for further guidance.

## Conclusion

Clinical research can be conducted using routinely measured and continuously recorded data from daily clinical practice. It is not limited to academic institutions—nephrologists working in community hospitals can also engage in meaningful clinical investigations. The key components of high-quality clinical research include: (1) a well-formulated clinical question, (2) appropriate data to address the question, and (3) the analytical skills necessary to interpret the data. This article provides an overview of each of these elements, with the aim of promoting high-quality clinical research in nephrology across Japan.

## Data Availability

Not applicable.

## Appendix 1. R script for eGFR calculation

2021 CKD-EPI creatinine-based eGFR equation with Japanese coefficient

2021 CKD-EPI creatinine and cystatin C-based eGFR equation with Japanese coefficient

JSN creatinine-based eGFR

JSN cystatin C-based eGFR

Example
